# HSP70 inhibits CHIP E3 ligase activity to maintain germline function in *Caenorhabditis elegans*

**DOI:** 10.1016/j.jbc.2024.107864

**Published:** 2024-10-09

**Authors:** Pankaj Thapa, Rupesh V. Chikale, Natalia A. Szulc, Maria-Teodora Pandrea, Agnieszka Sztyler, Khushboo Jaggi, Marta Niklewicz, Remigiusz A. Serwa, Thorsten Hoppe, Wojciech Pokrzywa

**Affiliations:** 1 Laboratory of Protein Metabolism, International Institute of Molecular and Cell Biology in Warsaw, Warsaw, Poland; 2 Department of Pharmaceutical and Biological Chemistry, School of Pharmacy, University College London, London, UK; 3 Institute for Genetics, University of Cologne, Cologne, Germany; 4 Cologne Excellence Cluster on Cellular Stress Responses in Aging-Associated Diseases (CECAD), University of Cologne, Cologne, Germany; 5 IMol Polish Academy of Sciences, Warsaw, Poland; 6 Center for Molecular Medicine Cologne (CMMC), Faculty of Medicine and University Hospital of Cologne, Cologne, Germany

**Keywords:** ubiquitin-proteasome system, proteostasis, HSP70, CHIP, germline integrity, C. elegans, proteotoxic stress, stress response mechanism, apoptosis, DAF-18/PTEN, lifespan

## Abstract

The ubiquitin-proteasome system is crucial for proteostasis, particularly during proteotoxic stress. The interaction between heat shock protein (HSP) 70 and the ubiquitin ligase CHIP plays a key role in this process. Our study investigates the *Caenorhabditis elegans* orthologs HSP-1 and CHN-1, demonstrating that HSP-1 binding decreases CHN-1 E3 ligase activity, aligning with the inhibitory effects observed in human HSP70–CHIP interactions. To explore the physiological significance of this inhibition, we utilized the HSP-1^EEYD^ mutant, which binds CHN-1 without reducing its activity, expressed in *C. elegans*. Our results reveal that the HSP-1–CHN-1 interaction is critical for maintaining germline integrity under heat stress by preventing excessive turnover of essential reproductive proteins. In HSP-1^EEYD^ nematodes, this protective mechanism is impaired, leading to disrupted stress-induced apoptosis, which is restored by CHN-1 depletion. Additionally, proteomic analysis identified DAF-18/PTEN as a potential CHN-1 substrate, which becomes destabilized when CHN-1 activity is not downregulated by HSP-1 during stress. Depleting DAF-18 significantly compromises the reproductive benefits observed from CHN-1 knockout in HSP-1^EEYD^ mutants, suggesting that the maintenance of DAF-18 plays a role in the observed phenotypes. These findings highlight the importance of HSP-1 in regulating CHN-1 E3 ligase activity to preserve germline function under stress conditions.

The ubiquitin-proteasome system is crucial for protein degradation, marking proteins for breakdown through ubiquitination. The E3 ubiquitin ligase CHIP (carboxy-terminus of Hsc70-interacting protein) plays a key role in this process by working with the chaperone network to ubiquitinate substrates (1). CHIP regulates proteins involved in stress responses, growth, and apoptosis, and its dysfunction is linked to diseases like neurodegeneration, cancer, and heart conditions (2, 3, 4). *Caenorhabditis elegans* expresses the CHIP ortholog CHN-1, which is structurally and functionally similar to human CHIP. CHN-1 is essential for muscle integrity by regulating the myosin chaperone UNC-45, impacting muscle development (5). It also influences longevity through the DAF-2/insulin/IGF-1 signaling pathway and methionine metabolism by controlling adenosylhomocysteinase turnover, connecting protein quality control with metabolic regulation (6, 7). CHIP/CHN-1, a dimeric protein, features an N-terminal tetratricopeptide repeat (TPR) domain, a central coiled-coil domain for dimerization, and a C-terminal U-box domain essential for its ubiquitin transfer activity (8). The TPR domain acts as a regulatory switch, interacting with heat shock protein 70 (HSP70) and HSP90 chaperones *via* their C-terminal EEVD motif, maintaining CHIP/CHN-1 in an autoinhibited conformation (7, 8, 9, 10). Prior research has illuminated the HSP70 role in attenuating CHIP ubiquitination activity in relation to proteins like Smad1/5 and α-synuclein (11, 12). The HSP70 C-terminal peptide (GPTIEEVD) alone can inhibit CHIP-mediated ubiquitination of substrates like p53 and IRF-1 by binding to the TPR domain (10). Our recent work using *C. elegans* orthologs of HSP70 and CHIP—HSP-1 and CHN-1—respectively, showed that HSP-1 interaction with CHN-1 TPR and U-box domains stabilizes the auto-inhibited CHN-1 dimer (7). We also found that mutating HSP-1 C-terminal EEVD motif to EEYD eliminates its inhibitory effect on CHN-1 ubiquitination activity *in vitro*, though the biological significance of this inhibition remains unclear.

We hypothesized that the interaction between HSP-1 and CHN-1 during proteotoxic stress modulates CHN-1/CHIP activity by restricting the ubiquitination of key substrates. To test this, we conducted molecular dynamics simulations to compare the binding and conformational effects of HSP-1 C-terminal decapeptide variants, focusing on the EEYD motif *versus* the wildtype EEVD motif. These simulations informed our *C. elegans* experiments, revealing that HSP-1 suppression of CHN-1 is critical for maintaining germline integrity under heat stress (HS). The HSP-1^EEYD^ mutation, which disrupts this inhibition, leads to the loss of essential germline proteins—some known CHIP/CHN-1 targets—resulting in reduced progeny survival at elevated temperatures. This disruption impairs stress-induced apoptosis, essential for removing damaged germ cells and maintaining germline homeostasis (13). Additionally, we identified DAF-18, the *C. elegans* PTEN ortholog (14), as a significant CHN-1 substrate. Stabilization of DAF-18 is crucial for the reproductive benefits and lifespan extension seen in HSP-1^EEYD^ mutants lacking functional CHN-1. Overall, our findings highlight HSP-1 role in modulating CHN-1 activity, acting as a safeguard to protect the germline proteome from excessive CHN-1-mediated degradation.

## Results

### Molecular dynamics uncover altered CHN-1 conformation upon interaction with GPTIEEV/YD peptides

**Figure 1:**
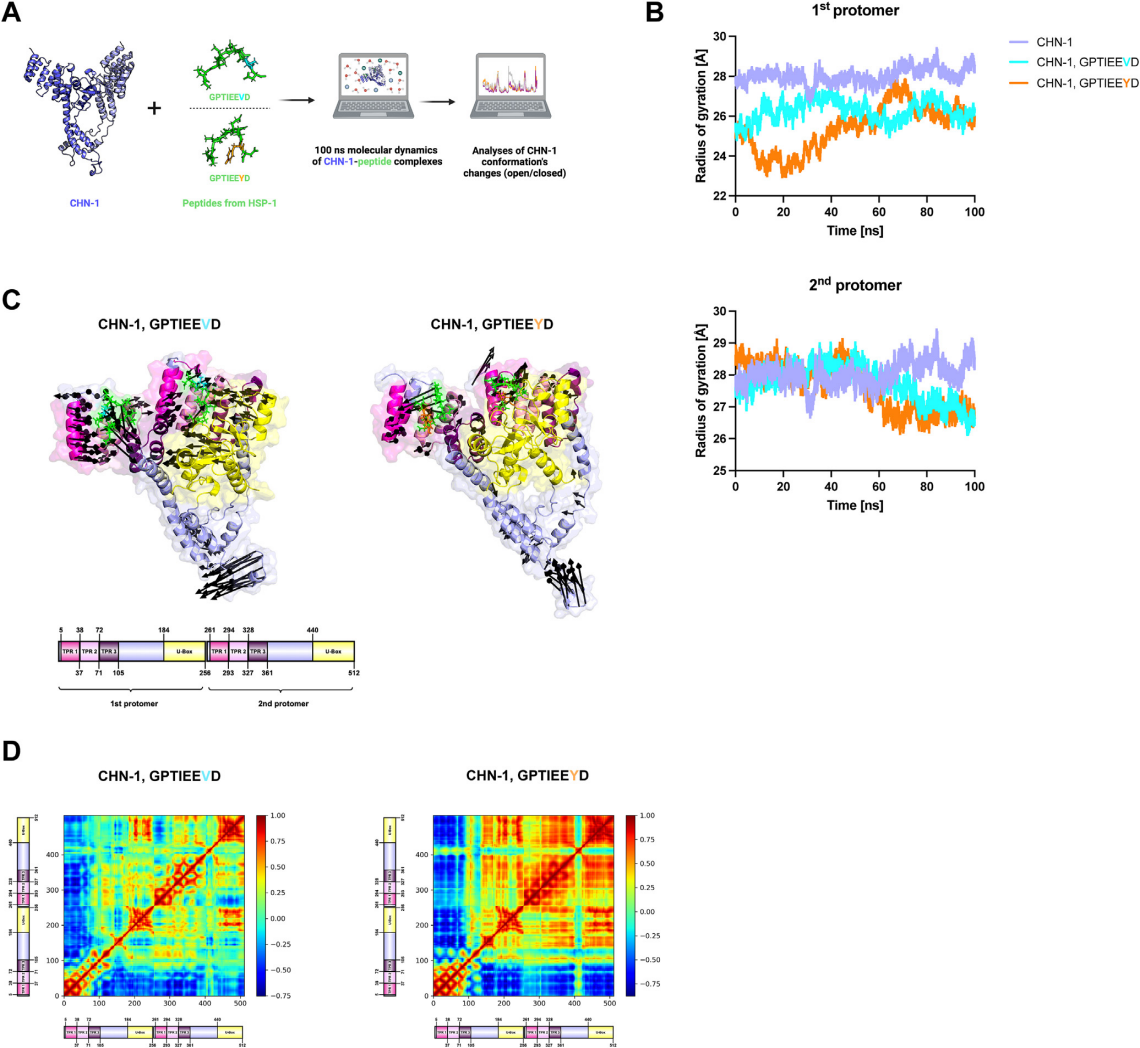
**CHN-1 exhibits altered conformations upon interaction with GPTIEEV/YD peptides.***A*, illustration of the molecular dynamics strategy, showcasing the CHN-1 dimer to explore conformational alterations caused by GPTIEEV/YD peptides. *B*, graph showing the radius of gyration for both CHN-1 protomers, with GPTIEEV/YD peptides and alone, illustrating structural compactness over time. *C*, normal mode analysis showing flexible domain movements in CHN-1 complexes, with *black arrows* highlighting the motion directions across 100 ns trajectories. An overview of CHN-1 dimer domain organization is provided below. *D*, cross-correlation matrix depicting C-alpha backbone movements relative to their mean position in CHN-1, with a heat map scale showing correlated (*red*) and anticorrelated (*blue*) motions along with an illustration of dimer domain organization. TPR, tetratricopeptide repeat.

Our prior investigation into the auto-ubiquitination activity of CHN-1 *in vitro* revealed that a valine (V) to tyrosine (Y) mutation of the C-terminal EEVD motif of HSP-1 negates its suppressive effect (7). Thus, to investigate whether the binding of peptides containing EEVD *versus* EEYD induces conformational changes of CHN-1 and to what extent, we utilized molecular dynamic simulations (Fig.1*A*). Our results indicated that the CHN-1 dimer alone undergoes dynamic changes, achieving a stable conformation after ∼70 ns of simulation, as corroborated by the RMSD plot (Fig. S1*A*). Interaction with HSP-1 peptide variants notably reduced CHN-1 lability, particularly near the second protomer of the TPR domain, as delineated on the root mean square fluctuation plot (Fig. S1*B*). Analysis of the radius of gyration also showed that HSP-1 peptides decrease intradomain flexibility and solvent exposure of CHN-1 (Fig. 1*B*). Clustering analysis revealed distinct conformations for the EEVD and EEYD peptides, with their most frequent poses showing significant differences in their interactions with CHN-1 (Fig. S1*C*). The EEYD peptide, which accounted for 39.92% of the trajectory, differed from the EEVD peptide by an RMSD of 1.709 Å and a centroid distance of 11.709 Å (Table S1), reflecting substantial structural divergence. Contact analysis also identified distinct sets of protein residues interacting with each peptide (Table S1). Additionally, molecular mechanics-generalized Born surface area analysis supported these findings, showing that the EEYD peptide binds more stably to CHN-1, with a significantly lower binding energy (ΔG_bind_ = −14.09 kcal/mol) compared to the EEVD peptide (ΔG_bind_ = −0.78 kcal/mol) (Table S2). These results suggest that the tyrosine mutation in EEYD leads to a more stable and distinct conformational state within the CHN-1 complex. Despite minimal individual protomer movement within the CHN-1 dimer during the simulation, we observed concerted, anticorrelated protomeric motions for the CHN-1 dimer with EEVD as compared to the complex with EEYD (Fig. 1, *C* and *D*). Principal component analysis of these movements revealed that CHN-1, when in complex with the GPTIEEYD peptide, displayed a compact structure around the central axis, whereas the GPTIEEVD peptide induced a more dispersed conformation of CHN-1 indicative of an energetically stable, autoinhibited state (Fig. S1*D*). Thus, the molecular dynamics simulations suggest that HSP-1^EEYD^ variant may affect CHN-1 activity by inducing a conformation that restricts its inhibitory state.

### HSP-1^EEYD^ worms exhibit oxidative resilience and heat sensitivity

**Figure 2:**
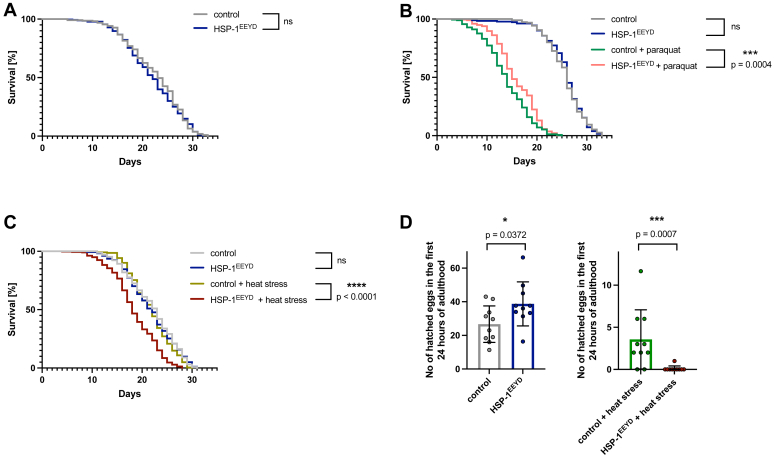
**Worms expressing HSP-1**^**EEYD**^**demonstrate resistance to oxidative stress and sensitivity to heat.***A*, survival curve of control and HSP-1^EEYD^ worms at 20 °C. Statistical significance was determined using Mantel-Cox log-rank test; the significance level per *p*-value is shown adjacent to the graph (ns—*p*-value > 0.05). The graph represents data from three biological repeats, n = 223 to 286 worms per strain. *B*, survival curve of control and HSP-1^EEYD^ worms with or without paraquat-induced oxidative stress. Statistical significance was determined using Mantel–Cox log-rank test; the stars denote the significance level per *p*-value and are shown adjacent to the graph (ns—*p*-value > 0.05). The graph represents data from three biological repeats, n = 204 to 216 worms per strain per condition. *C*, survival curve of control and HSP-1^EEYD^ worms exposed or not to HS. Statistical significance was determined using Mantel–Cox log-rank test; the *stars* denote the significance level per *p*-value and are shown adjacent to the graph (ns—*p*-value > 0.05). The graph represents data from three biological repeats, n = 227 to 317 worms per strain per condition. *D*, Bar plot of hatched eggs in the first 24 h of adulthood for control and HSP-1^EEYD^ worms with or without HS. Data from two repeats, 15 worms per strain per condition. Statistical significance was determined using Mann–Whitney test, unpaired, 2-tailed; the *stars* denote the significance level per *p*-value and are shown adjacent to the graph. The results are plotted as the mean ± S.D. Data analyzed with GraphPad Prism 10. HS, heat stress; HSP, heat shock protein.

Recognizing that HSP-1^EEYD^ binds CHN-1 without inhibiting its ubiquitination, we used this interaction to evaluate its functional significance in *C. elegans*. By employing CRISPR-Cas9 technology, we introduced a targeted V to Y substitution in the EEVD motif of HSP-1, creating the *hsp-1(syb5159)*, p.V639Y variant, known as HSP-1^EEYD^, to explore CHN-1-dependent phenotypes linked to this specific mutation. While RNAi-mediated depletion of HSP-1 is known to cause developmental defects (15, 16), we observed that nematodes expressing solely HSP-1^EEYD^ showed no early larval arrest, displayed no noticeable defects in adulthood, and demonstrated aging trajectories that are comparable to those observed in wildtype controls (Fig. 2*A*). This suggests that the HSP-1^EEYD^ mutation does not constitute a loss-of-function alteration under normal growth conditions. To elucidate the role of the EEVD motif in HSP-1 during impaired proteostasis, we subjected HSP-1^EEYD^ worms to different proteotoxic stress conditions. Initially, we assessed the adult worms oxidative stress resistance by chronically exposing them to 4 mM paraquat from the L4 larval stage (17, 18). The HSP-1^EEYD^ worms exhibited a significant enhancement in survival rates in comparison to the wildtype controls when treated with paraquat (Fig. 2*B*). Following this, we evaluated their response to prolonged HS. In this protocol, L4 larvae (grown at 20 °C) were exposed to an elevated temperature of 30 °C for 16 h. Subsequently, we measured their post-HS lifespan at 20 °C to assess their recovery. Contrasting with their increased resilience to oxidative stress, the HSP-1^EEYD^ worms demonstrated heightened sensitivity to HS, as evidenced by a shorter lifespan (Fig. 2*C*). Prompted by these findings, we examined the impact of HS on germ cell preservation and reproductive capacity in HSP-1^EEYD^ mutants. L4 larvae were subjected to 16 h of HS, after which the mutants, initially showing higher early adult brood size than controls, exhibited a significant reduction poststress (Fig. 2*D*). This underscores the reproductive system sensitivity to heat-induced proteotoxicity and highlights the critical role of wildtype HSP-1 in maintaining germline integrity under stress.

### Impaired CHN-1 inhibition by HSP-1^EEYD^ leads to reproductive and lifespan alterations under HS

**Figure 3:**
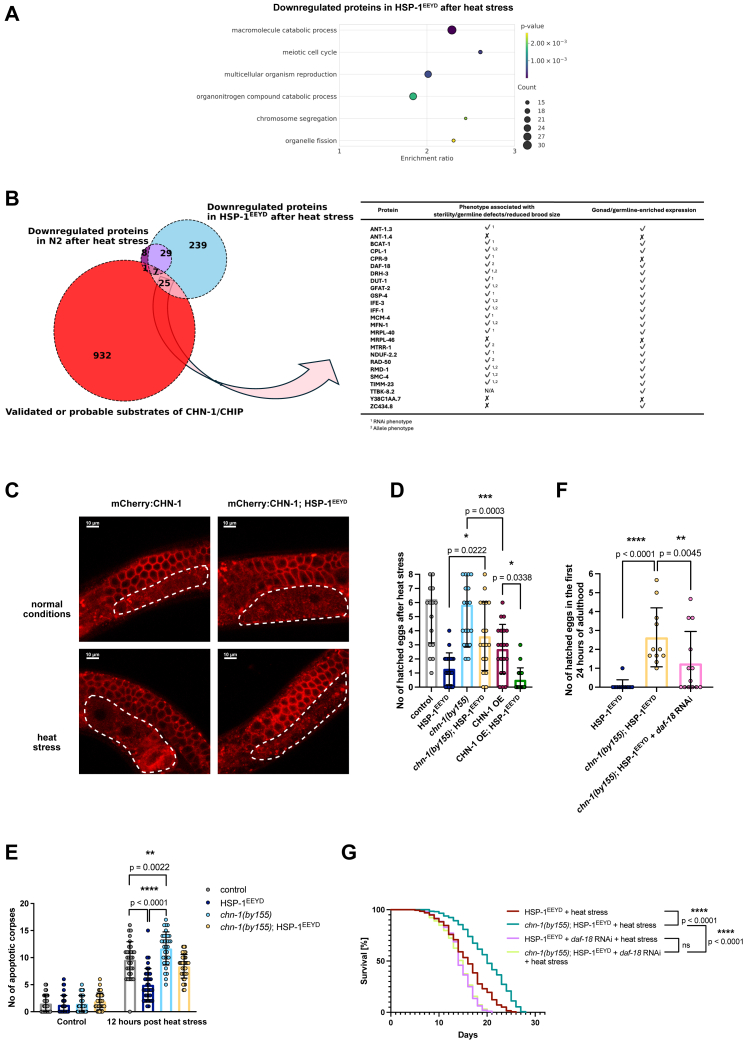
**Heat stress induces reproductive and lifespan changes through impaired CHN-1 inhibition by HSP-1**^**EEYD**^. *A*, GO biological process terms (nonredundant) found to be associated with proteins downregulated in HSP-1^EEYD^ worms under HS; all proteins detected in our proteomics analysis comprised a reference set. Overrepresentation analysis using the WebGestalt web server with default parameters (41). False discovery rate was controlled to 0.1 using the Benjamini–Hochberg method for multiple testing. *B*, Venn diagram depicting proteins that are significantly downregulated in both control and HSP-1^EEYD^ worms after HS, intersecting with CHN-1/CHIP substrates, as detailed in an adjacent table. *C*, confocal microscopy images showing the presence of mCherry::CHN-1 in the germline of control and HSP-1^EEYD^ worms post-HS. *Dashed lines* indicate embryos. *D*, bar plot of hatched eggs in the first 24 h post-HS across worm strains. Data from two repeats, 8 to 10 worms per strain per condition. Significance determined by 1-way ANOVA; the *stars* denote the significance level per *p*-value. The results are plotted as the mean ± S.D. *E*, plot showing germline apoptosis 12 h post-HS (16h at 30 °C) and under control conditions across indicated worm strains. Data were analyzed using 2-way ANOVA. Statistical significance was determined using Tukey’s test; *p*-values are shown on the graph. Data are from three biological replicates (n = 10–15 worms/condition per replicate). The results are plotted as the mean ± S.D. *F*, bar plot of hatched eggs within 24 h post-HS for worm strains following DAF-18 RNAi. The graph represents data from three biological repeats each containing 7 to 8 worms per strain per condition. Data were analyzed using 2-way ANOVA. Statistical significance was determined using Tukey's multiple comparisons test; the *stars* denote the significance level per *p*-value. The results are plotted as the mean ± S.D. *G*, survival curve of HSP-1^EEYD^ and *chn-1(by155)*, HSP-1^EEYD^ with or without HS and DAF-18 RNAi. Statistical significance was determined using Mantel–Cox log-rank test; the stars denote the significance level per *p*-value (ns—*p*-value > 0.05). The graph represents data from two biological repeats, n = 193 to 267 worms per strain per condition. Data analyzed with GraphPad Prism 10. GO, gene ontology; HS, heat stress; HSP, heat shock protein.

Given that the *chn-1(by155)* loss-of-function mutation does not affect the oxidative stress resistance of HSP-1EEYD worms (Fig. S2*A*), we investigated their sensitivity to HS, particularly in the reproductive system. We hypothesized that the EEYD mutation fails to downregulate CHN-1, leading to accelerated degradation of proteins essential for reproduction under HS. To test this, we performed proteomic analysis comparing protein profiles of HSP-1^EEYD^ and wildtype worms, with and without 16-h HS at the L4 stage. We quantified 5356 protein groups across four conditions, revealing significant differences in protein levels between wildtype and HSP-1^EEYD^ worms in response to HS (Table S3). The levels of 76 protein groups were increased and 44 decreased in the former, and 325 protein groups were increased and 279 decreased in the latter. We analyzed the intersections of the downregulated protein in these two worm populations to dissect the specific pathways affected by the interplay of HS and HSP-1^EEYD^ expression. Next, drawing upon both our prior work and existing literature (7, 19, 20, 21, 22), as well as interactome databases (23, 24), we compiled a comprehensive list of CHN-1/CHIP interactors and substrates (Table S4). The analysis of over-represented Gene Ontology terms for proteins that are downregulated in HSP-1^EEYD^ worms upon HS indicated a significant enrichment of pathways associated with catabolism and reproduction (Fig. 3*A*). Subsequently, we conducted a comparative study on proteins that are downregulated in HSP-1^EEYD^ and wildtype worms under HS by overlapping them with CHN-1/CHIP interactors and substrates. This analysis revealed a significantly higher number of downregulated CHN-1 substrates in HSP-1^EEYD^ (25 CHN-1 substrates/interactors, Table S5) than in wildtype worms (eight CHN-1 substrates/interactors), while the level of CHN-1 itself remained unchanged (Fig. 3*B*, Tables S3, S5). Notably, the majority of these proteins were predominantly expressed in the germline or gonads and implicated in germline defects, sterility, or reduced brood size according to RNAi depletion or allele-specific studies (Fig. 3*B*, Table S5).

Next, by utilizing HSP-1^EEYD^ worms, we aimed to discern the effects of HSP-1 possible failure to suppress CHN-1 germline function. To achieve this, we employed the CRISPR/Cas9 methodology to generate endogenous CHN-1 tagged with mCherry (mCherry::CHN-1), which allowed us to confirm its prominent presence in reproductive tissues, namely germ cells, oocytes, and spermatheca (Fig. 3*C*). Following HS, mCherry::CHN-1 distribution in HSP-1^EEYD^ hermaphrodite gonads was comparable to controls, yet these worms displayed significant reproductive impairment, as evidenced by a complete lack of oocytes. Eliminating CHN-1 (*chn-1(by155)* loss of function allele) reinstated oocyte formation in HSP-1^EEYD^ mutants, enabling them to produce eggs post-HS, unlike their HSP-1^EEYD^ counterparts with active CHN-1 (Figs. S2*B* and 3*D*). Consequently, overexpression of CHN-1 (CHN-1 OE) under its native promoter (6) diminished egg production in both wildtype and HSP-1^EEYD^ strains (Fig. 3*D*), with immunostaining confirming an increase in CHN-1 levels in the gonads of the CHN-1 OE strain (Fig. S2*C*). HSP-1^EEYD^ worms at 20 °C showed an increase in the number of hatched eggs compared to controls; this effect was largely independent of CHN-1, as the absence of CHN-1 (*chn-1(by155)*) had no effect, while overexpression (CHN-1 OE) slightly influenced the outcome (Fig. S2*D*). These findings suggest that HSP-1 ability to moderate CHN-1 activity on specific germline proteins during HS is crucial for maintaining reproductive health.

In *C. elegans*, apoptosis acts as a vital quality control mechanism by eliminating damaged or surplus germ cells, thereby maintaining gonadal homeostasis, especially under heat shock conditions that increase apoptotic germ cell incidence (13). We observed these apoptotic changes using differential interference contrast, identifying apoptotic corpses as condensed chromatin bodies with highly refractile properties within the gonads. Following exposure to 30 °C for 16 h at the late L4 stage, there was a notable increase in apoptotic germ cells, scored 12 h post-heat shock. In HSP-1 mutants, the incidence of apoptotic germ cells was reduced, suggesting that HSP-1 might influence apoptosis through its regulation of CHN-1. Notably, CHN-1 knockout (*chn-1(by155*)) restored apoptotic levels under HS in HSP-1^EEYD^ worms, underscoring the modulatory role of the HSP-1-CHN-1 interaction in apoptosis during environmental stress (Fig. 3*E*).

Given the significant effects of CHN-1 deletion on reproductive function and lifespan in HSP-1^EEYD^ mutants exposed to 16-h HS (Figs. 2*C* and S2*E*), we explored potential targets involved, with DAF-18 standing out. DAF-18, the *C. elegans* ortholog of human PTEN, is a key regulator of the insulin/IGF-1 signaling pathway, crucial for longevity and stress resistance (14). Human CHIP, the mammalian CHN-1 ortholog, targets PTEN for proteasomal degradation, suggesting a conserved mechanism (25). In our study, DAF-18 depletion significantly reduced the reproductive and lifespan benefits seen after CHN-1 knockout in HSP-1^EEYD^ mutants (Fig. 3, *F* and *G*). This implies that the improved outcomes in these mutants may be due to the preservation of DAF-18, which would otherwise be excessively degraded under uncontrolled CHN-1 activity. Our findings highlight that HSP-1 suppression of CHN-1 ubiquitination is vital for maintaining germline integrity and overall health during HS, underscoring the importance of DAF-18 stabilization in these protective processes.

## Discussion

Our study elucidates why HSP-1 reduces CHN-1 activity. Molecular dynamics simulations demonstrated that the HSP-1EEYD variant, which fails to inhibit CHN-1, induces distinct conformational changes. In *C. elegans*, this results in heightened sensitivity to HS, particularly affecting the reproductive system. Proteomic analyses revealed that insufficient HSP-1 inhibition in the mutant destabilizes key CHN-1/CHIP substrates, impairing germline function. Localization studies confirmed CHN-1 presence in the germline, where its genetic ablation improved oocyte survival in HSP-1EEYD animals, while increased CHN-1 expression exacerbated reproductive defects. These findings highlight the crucial role of HSP-1 in regulating CHN-1 during thermal stress, though further assays are required to fully understand the underlying mechanisms. In *C. elegans*, apoptosis serves as a quality control mechanism to eliminate damaged or surplus germ cells, maintaining gonadal homeostasis (26, 27). This physiological process is complemented by stress-induced apoptosis pathways, which are activated in response to environmental challenges such as heat, oxidative stress, and pathogen infection (28, 29, 30, 31). Our findings suggest that HSP-1 mediates apoptosis under HS by modulating CHN-1 activity, likely reducing its processivity as an E3 ligase. This interaction is crucial for germ cell survival, as the HSP-1^EEYD^ mutation restricts stress-induced apoptosis, compromising germline integrity. While speculative, CHN-1 substrates potentially regulated by HSP-1 include DUT-1, a dUTPase preventing uracil misincorporation into DNA, and CPL-1, a lysosomal cathepsin involved in apoptotic cell clearance. The downregulation of these proteins *via* RNA interference leads to phenotypes associated with germ-cell apoptosis (32, 33). Additionally, the sterility observed in HSP-1^EEYD^ mutants under HS may partly stem from disruptions in P-granule function, essential for mRNA surveillance and germ cell development. Notably, mutations affecting P-granule components have been associated with sterility under high temperatures (34, 35). The detected decrease in the level of proteins such as DRH-3, a Dicer-related helicase and a potential CHN-1 substrate, point to potential disturbances in germ granule dynamics contributing to the sterility phenotype of HSP-1^EEYD^ mutants under HS (36, 37). However, given that germ granule defects are typically reported at temperatures above 30 °C, higher than those in our study, the sterility we observed likely involves broader deregulation of multiple CHN-1 targets, making it difficult to isolate the impact of individual substrates on fertility (38).

Among the targets identified in our proteomic studies, DAF-18, the *C. elegans* ortholog of PTEN, was found to play a significant role. DAF-18 is a phosphatase crucial to the insulin/IGF-1 signaling pathway, which influences both longevity and stress resistance in *C. elegans* (36, 37). Our results show that HSP-1 regulation of CHN-1 is crucial for maintaining DAF-18 levels, and its downregulation in HSP-1^EEYD^ worms likely contributes to reduced lifespan and fertility under HS. We speculate that unchecked CHN-1 activity disrupts the balance of protein degradation, affecting both immediate stress responses and long-term health. This highlights the complex interplay between proteostasis and signaling pathways in regulating lifespan and stress adaptation. Identifying additional CHN-1 substrates and understanding how HSP-1 modulates their degradation will deepen our understanding of aging and stress resistance. Further investigation is needed to explore how the altered CHN-1 conformation affects ubiquitination targets and signaling pathways. Additionally, the HSP-1^EEYD^ mutation may influence interactions with other TPR domain proteins, potentially affecting stress responses and lifespan through a broader range of protein interactions.

## Experimental procedures

Detailed experimental procedures, including protocols for microscopy, proteomics, and *in silico* analyses, can be found in the supporting information.

### Worm maintenance and strains

Worms were maintained on nematode growth medium (NGM) plates seeded with OP50 *Escherichia coli* bacteria at 20 °C unless otherwise stated (39). A list of all *C. elegans* strains used in the study is provided in Table S7.

The *hsp-1(syb5159)* strain was generated by SunyBiotech using CRISPR/Cas9 technology. A single guide RNA was designed to target the sequence CCAACGATCGAGGAGGTCGACTA within the *hsp-1* gene. To introduce the EEYD mutation, the repair template oligonucleotide sequence used was as follows:

CACCAGGTGCTGCTCCAGGAGGAGCCGCCGGAGGAGCTGGAGGACCAACGAT**T**GAGGAGTACGACTAAttatttatcttcttttttgatctcggtttttatctttattctct.

In this sequence, the synonymous mutation introduced to prevent recutting by the CRISPR/Cas9 system is highlighted in bold and underlined.

For validation, the following primers were used for amplification and sequencing of the target region:

Forward Primer: GAGAAATACAAGGCTGACGA.

Reverse Primer: CTGCACCGCCTATGTATTAA.

After generating the hsp-1(syb5159) strain, it was outcrossed with N2 wildtype worms for four generations.

### Lifespan assay

All lifespan measurements were done from the L4 stage at 20 °C on NGM plates containing 400 μM FUdR as described previously (40). Briefly, during lifespan measurements, worms were scored daily for movement and pharyngeal pumping until death. Animals that crawled off the plate or exhibited baggy phenotypes were censored from the experiment. Survival analysis under oxidative stress was performed following the transfer of L4 staged worm strains to paraquat (4 mM) and FUdR (400 μM) containing plates. Lifespan comparison for HS recovery studies was performed by treating the control and mutant worms with or without HS, *i.e.,* 16-h 30 °C treatment at the L4 stage. Immediately after the treatment, the worms were transferred to 20 °C on NGM plates with FUdR (400 μM) and scored daily as described above. Statistical data of individual lifespan experiments are presented in Table S6. The experiments were not randomized. No statistical methods were used to predetermine the sample size. The investigators were blinded to allocation during experiments.

### Egg hatching analysis

The worms were maintained at 20 °C until they reached the L4 stage. The animals were then subjected to HS for 16 h at 30 °C. A single worm from each strain was then separately transferred to new NGM plates and incubated for 24 h at 20 °C. The hatched eggs were then counted. Statistical data of individual experiment are presented in Table S6.

## Data availability

The mass spectrometry proteomics data were deposited to the ProteomeXchange Consortium *via* the PRIDE partner repository with the dataset identifier PXD048200. The raw data and simulations have been deposited in Zenodo and are accessible at the provided https://doi.org/10.5281/zenodo.13837731.

## Supporting information

This article contains Supporting information.

## Conflict of interest

The authors declare no conflict of interest with the contents of this article.
