# Supplementary figures and images for: HSP70 inhibits CHIP E3 ligase activity to maintain germline function in Caenorhabditis elegans
Source: J Biol Chem. 2024 Oct 9;300(11):107864. doi: 10.1016/j.jbc.2024.107864 (PMC11567022; doi:10.1016/j.jbc.2024.107864)

A

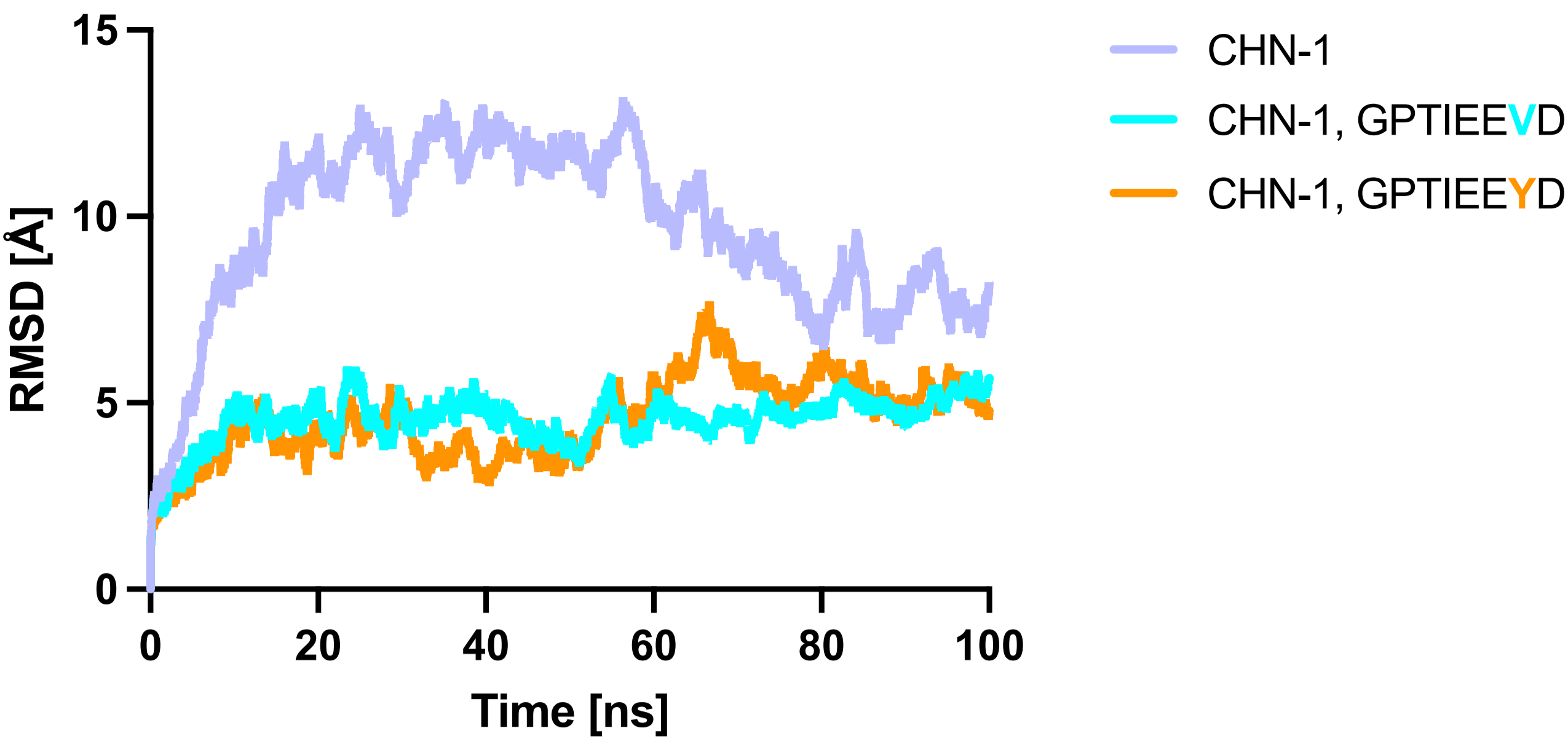

B

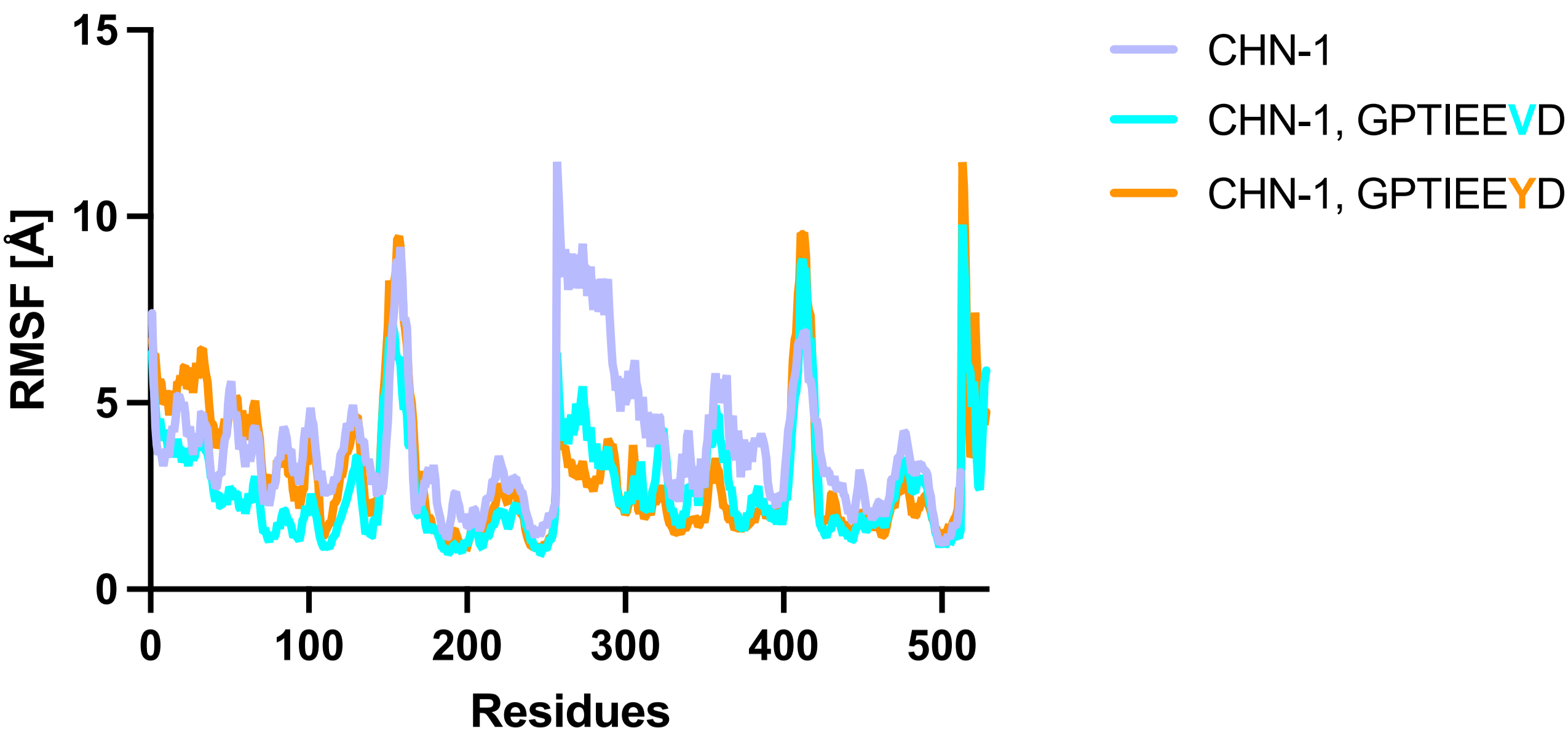

C

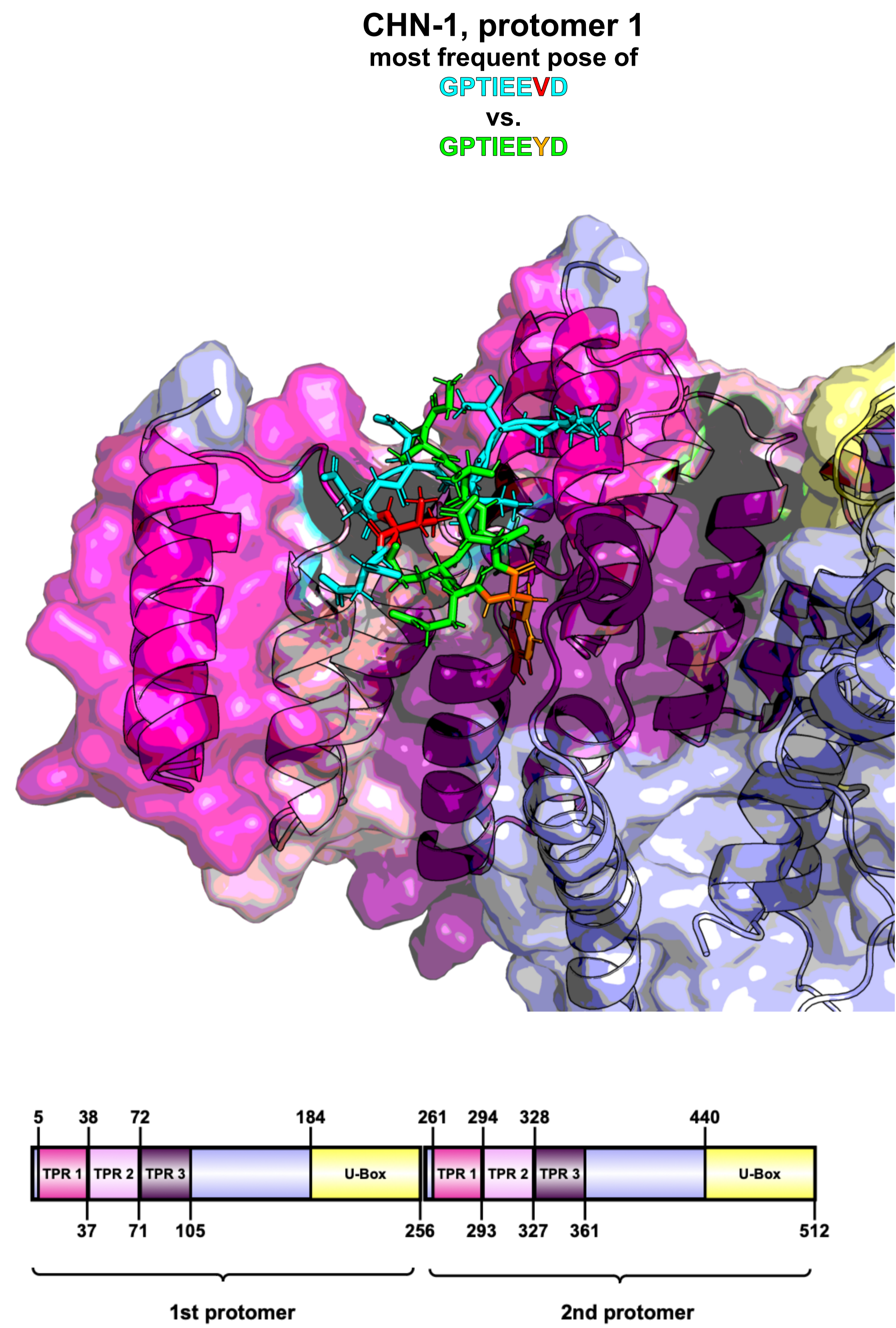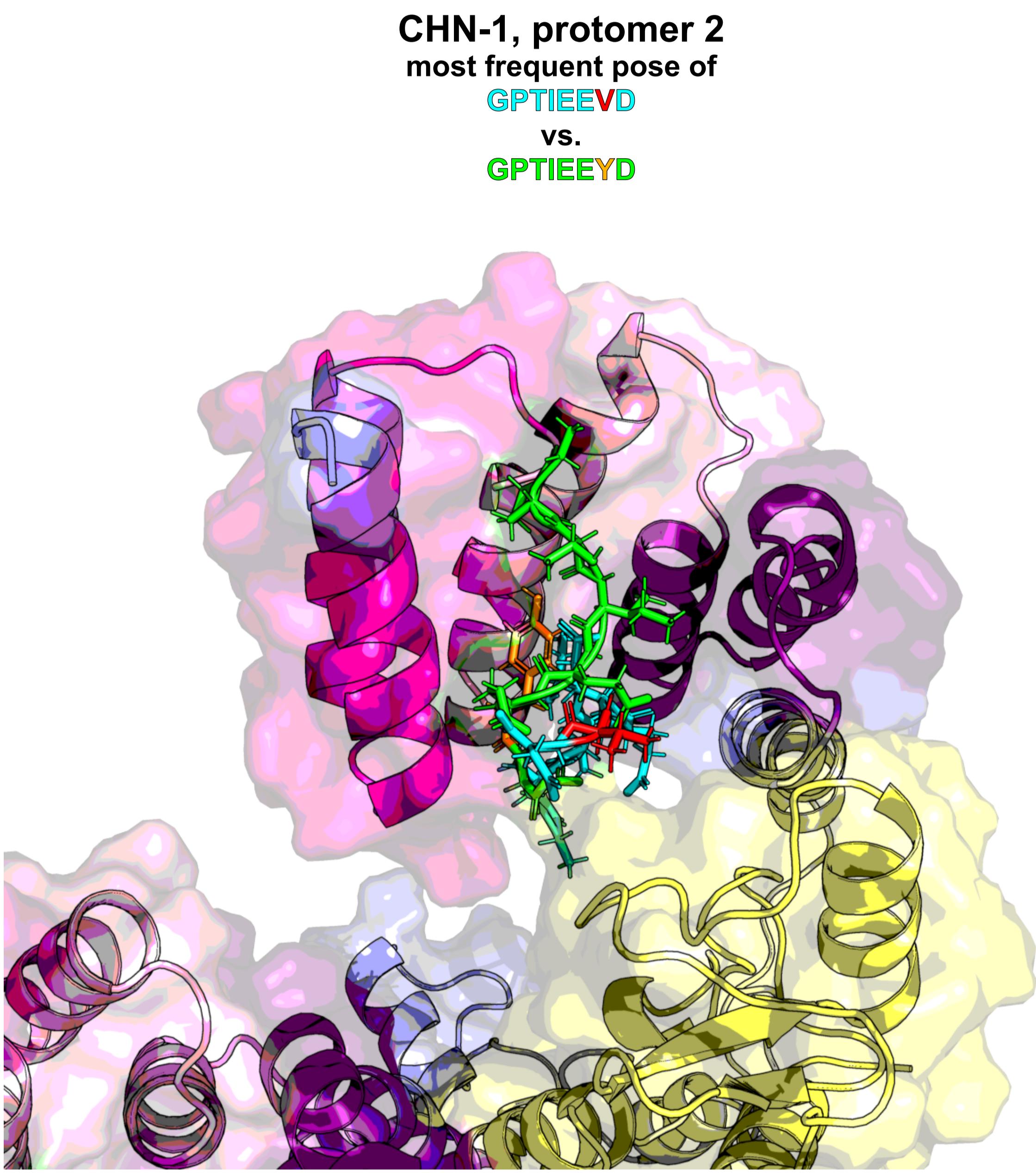

D

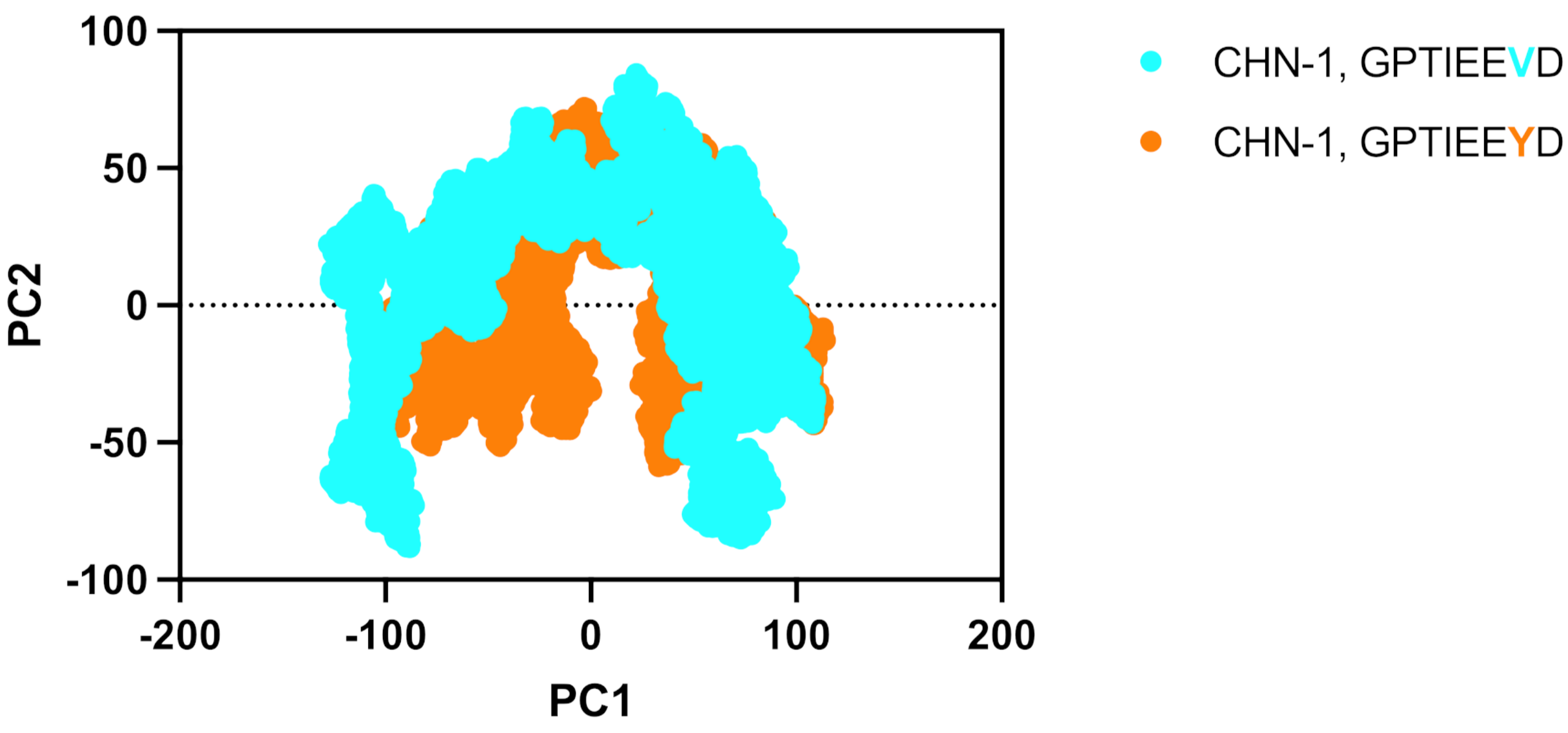

Supplement: Supplemental Figure S1 [file mmc1.pdf]

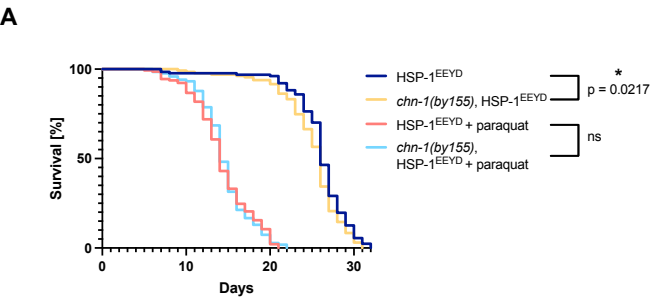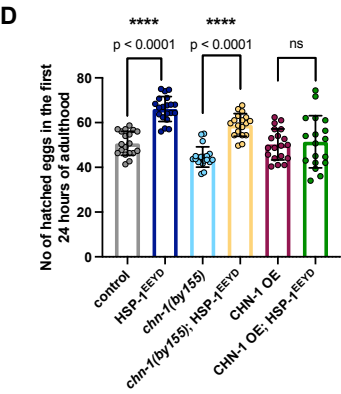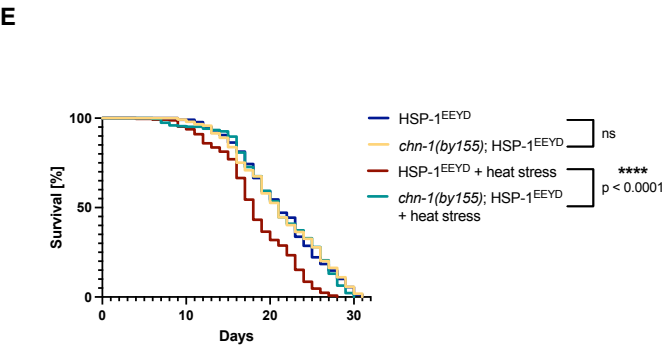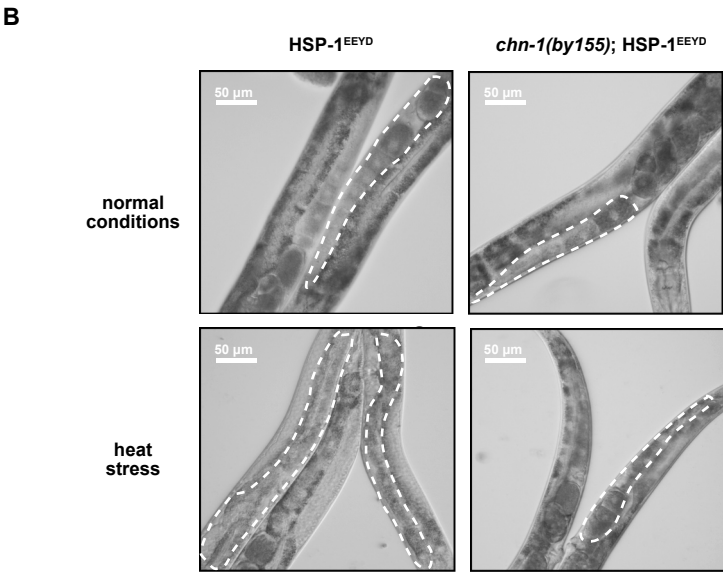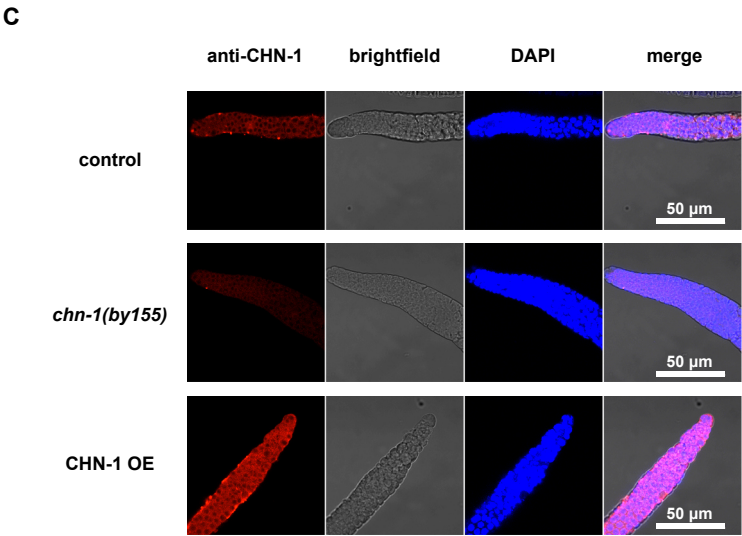

Supplement: Supplemental Figure S2 [file mmc2.pdf]

control

HSP-1<sup>EEYD</sup>

control

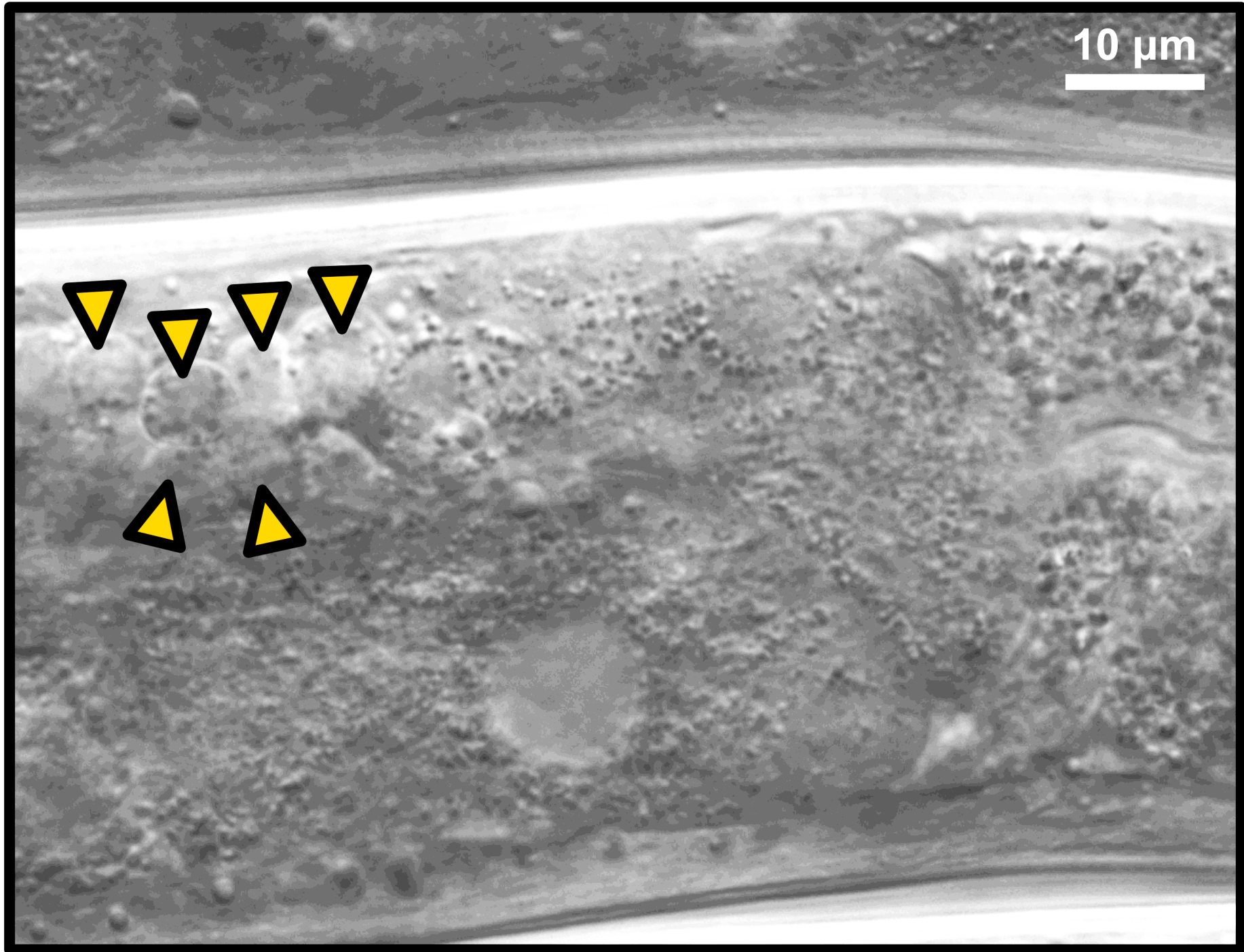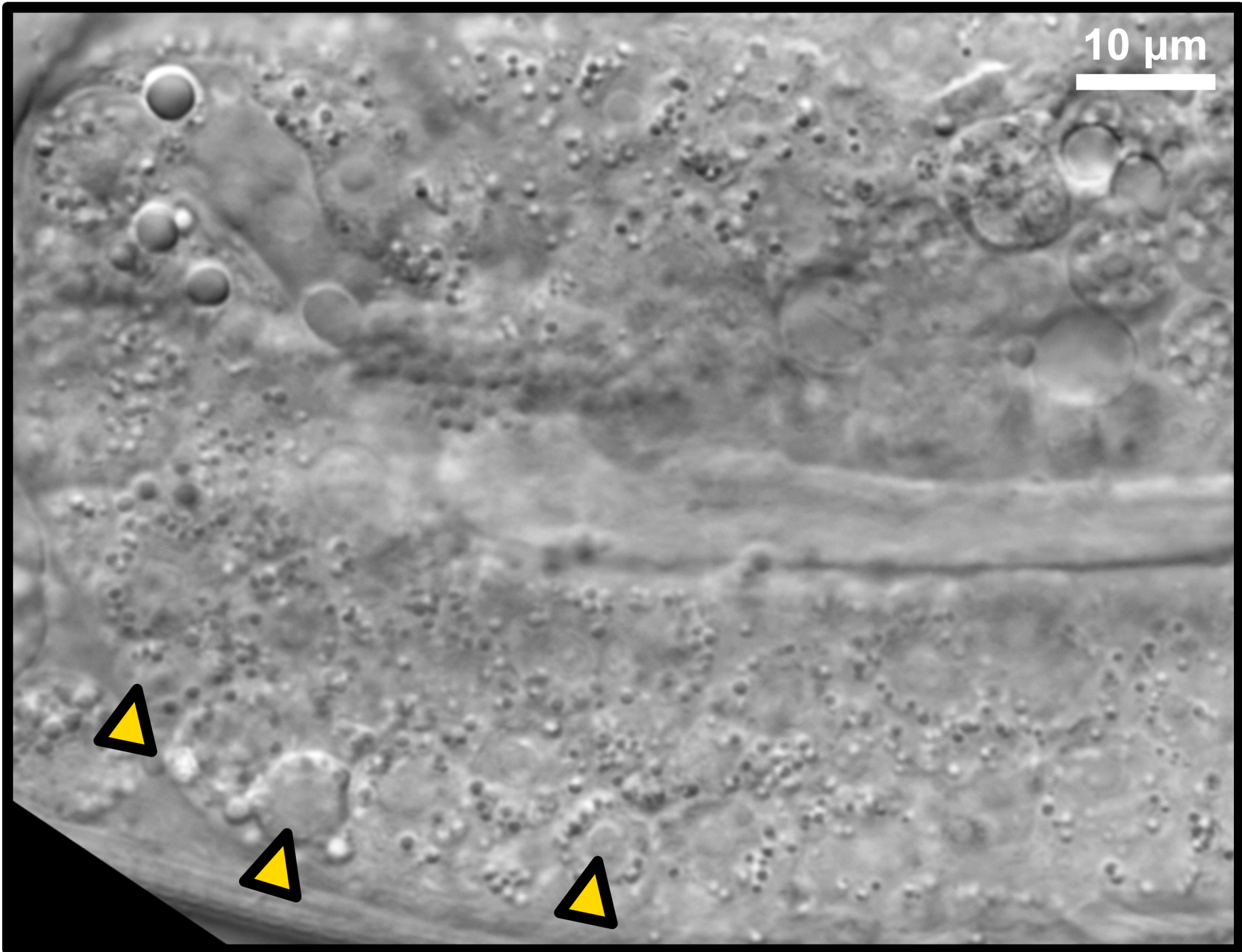

*chn-1(by155)*

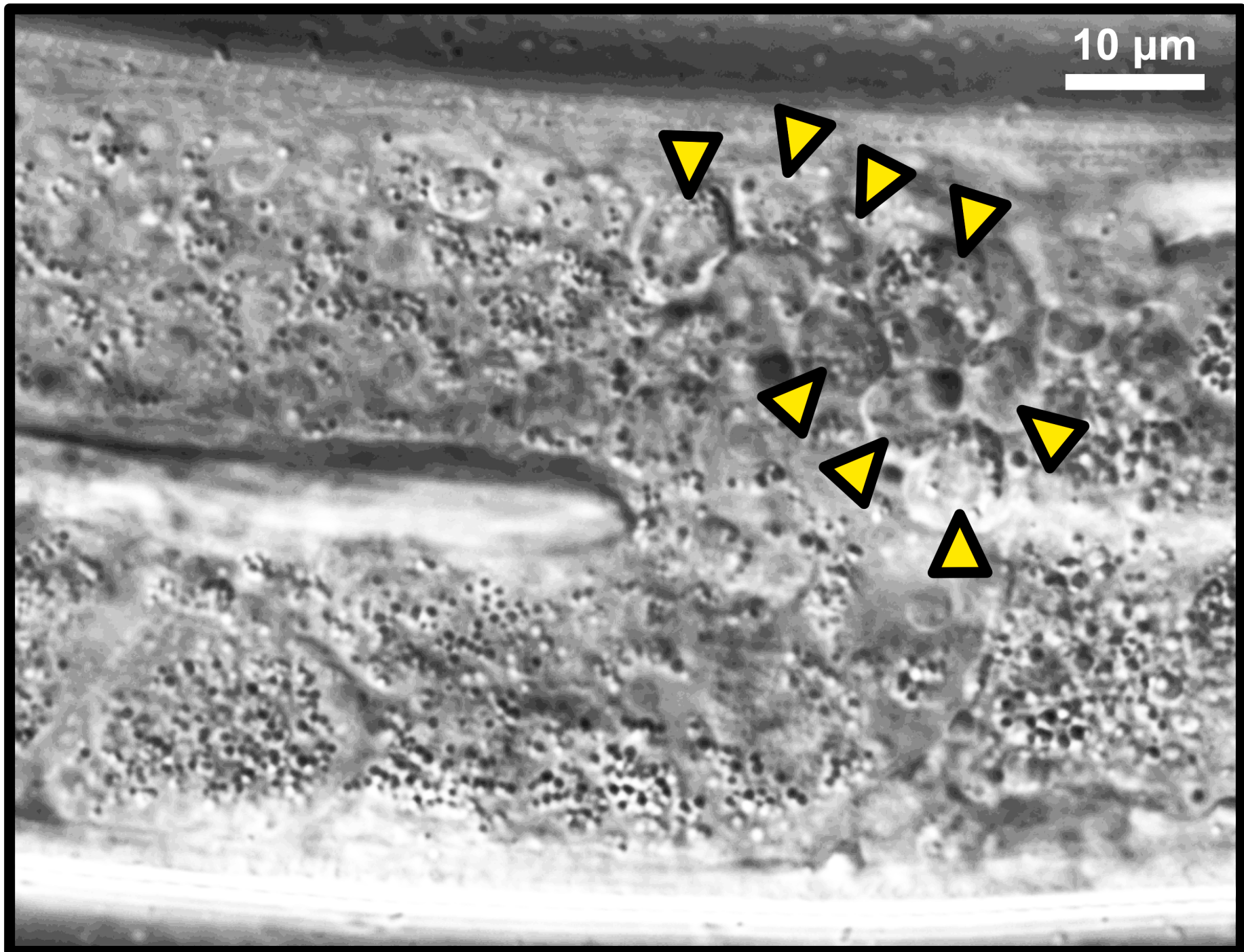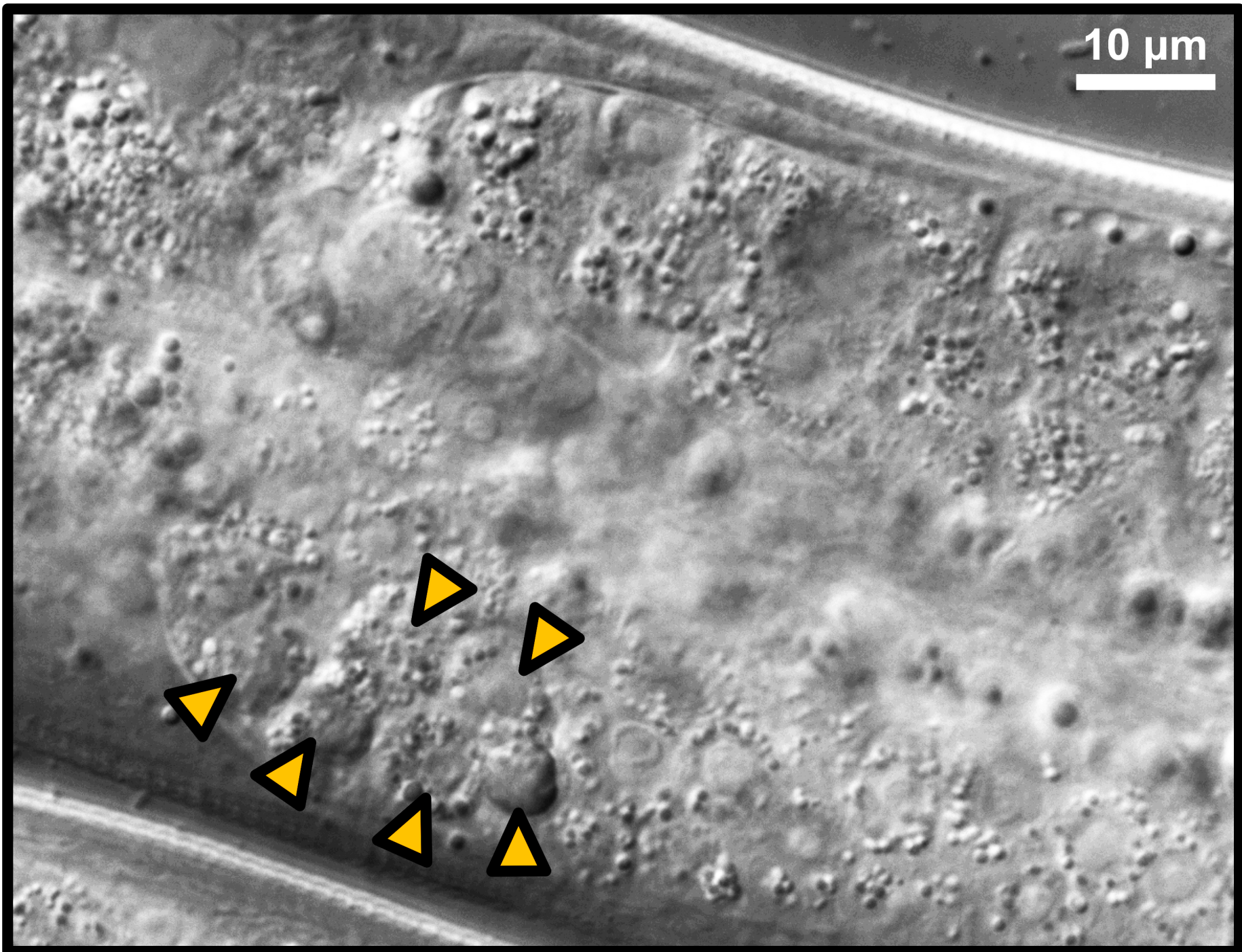

Supplement: Supplemental Figure S3 [file mmc3.pdf]
