# Supplementary material for: HSP70 inhibits CHIP E3 ligase activity to maintain germline function in Caenorhabditis elegans
Source: J Biol Chem. 2024 Oct 9;300(11):107864. doi: 10.1016/j.jbc.2024.107864 (PMC11567022; doi:10.1016/j.jbc.2024.107864)
Supplement: Supporting information [file mmc4.pdf]

HSP70 inhibits CHIP E3 ligase activity to maintain germline function in *Caenorhabditis elegans*

Pankaj Thapa, Rupesh V Chikale, Natalia A. Szulc, Maria-Teodora Pandrea, Agnieszka Sztyler, Khushboo Jaggi, Marta Niklewicz, Remigiusz A. Serwa, Thorsten Hoppe, Wojciech Pokrzywa

<u>Materials included:</u>

Supporting experimental procedures

Supplemental figure S1

Supplemental figure S2

Supplemental figure S3

Supplemental table S1

Supplemental table S2

Supporting experimental procedures

Molecular dynamics simulations

The CHN-1 model construction was initiated on the Swiss Model server (1), employing a structure with PDB ID: 2C2L (2) as a template. Subsequent model preprocessing was performed using Schrodinger Glide (3). It involved protein preparation steps where the protonation state was corrected by adding missing hydrogens and correction in the orientation of histidine, asparagine, and glutamine side-chain. The partial charge assignment was performed using the OPLS-2005 force field, and energy minimization was performed to relieve any strain energies in the system after protonation using GLIDE workflow. The model next underwent molecular dynamics simulations leveraging AMBER18 software (4–6) on a system equipped with CUDA-capable graphics processing units. The CHN-1 model was prepared in the program Xleap in AmberTools20 (7). The model was placed in a truncated octahedral solvent box, and an explicit solvent approach was adopted using the TIP3P water model (8). A sufficient number of counter ions Na+ and Cl− were added to neutralize the simulation system, and 0.1 M of ionic strength was achieved. The system was adjusted at 7.4 pH. The model parameterization was done using the FF14SB force field (9).

MD simulations were run at 1 atm constant pressure (Monte Carlo Barostat) at 300 K constant temperature (Langevin Thermostat) with a collision frequency of 2ps-1 and the volume exchange was performed every 100 fs. SHAKE was used with an integration step of 2 fs for the simulation of hydrogen bonds (10). For long-range electrostatic interaction, particle mesh ewald methods (PME) were applied, and for the short-range interactions, a cutoff of 8 Å was used (11). Prior to the molecular dynamics production phase, the model underwent several preparatory steps to ensure relaxation and physiological mimicry of the protein complex. The preparation phase consisted of (i) an initial 5 ns of water minimization, (ii) followed by 5 ns of solute/protein minimization with applied restraints, (iii) a combined 5 ns minimization of both water and solute and (iv) capped with 5 ns of simulated annealing.

The equilibration process comprised two phases: NVT equilibration for 10 ns, followed by NPT equilibration for another 10 ns. Subsequently, a 100 ns long structural simulation was conducted. Post-simulation, coordinate trajectories, and other relevant data files were analyzed using CPPTRAJ (12) to calculate radius of gyration, RMSF and RMSD values presented in Fig 1 and Fig S1.

GPTIEEV/YD peptides docking into the last snapshot of CHN-1, as derived from the molecular dynamics simulation of CHN-1 alone, was conducted using the Bioluminate tool from Schrodinger (13). A docking grid was established around the EEVD motif from the Hsp90 peptide of the 2C2L structure. Subsequently, the final structures were refined via energy minimization and integrated into the above-described molecular dynamics simulation pipeline devised for creating the CHN-1 model.

Clustering of the peptide backbone conformations was performed using the k-means algorithm implemented in MDAnalysis (14, 15). The clustering was conducted with a predefined number of clusters (k=3) to capture the dominant conformational states. Every 5th frame from the 100 ns trajectory was sampled to ensure a broad representation of the peptide's dynamics. The most frequent cluster for each peptide was identified (table below) and selected for subsequent analyses.

Clustering results for the peptide conformations during the 100 ns molecular dynamics simulations. The analysis was conducted using the k-means algorithm with three clusters (k=3), and every 5th frame from the trajectory was sampled. The most frequent cluster, which was chosen for subsequent structural comparisons and analysis, is bolded.

Peptide variant	Protomer	Cluster	Number of Frames	Percentage of Trajectory
**EEYD**	Protomer 1	Cluster 0	2216	27.70%
		Cluster 1	2590	32.38%
		**Cluster 2**	**3194**	**39.92%**
	Protomer 2	Cluster 0	2569	32.11%
		**Cluster 1**	**2739**	**34.24%**
		Cluster 2	2692	33.65%
**EEVD**	Protomer 1	**Cluster 0**	**3410**	**42.62%**
		Cluster 1	2150	26.88%
		Cluster 2	2440	30.50%
	Protomer 2	Cluster 0	1451	18.14%
		Cluster 1	1468	18.35%
		**Cluster 2**	**5081**	**63.51%**

The MM-GBSA analysis was performed using the Amber18 and AmberTools18 programs (13, 16) on the 100 ns trajectories, covering the final 10,000 frames of CHN-1 with EEVD and EEYD, respectively.

RNAi treatment

RNA interference (RNAi) was performed using the feeding method as described by Kamath et al., 2001(17). L1 larvae were placed on NGM plates containing IPTG and ampicillin, seeded with *E. coli* HT115(DE3) expressing double-stranded RNA (dsRNA). The larvae were grown until the L4 stage before undergoing heat shock and subsequent apoptotic corpse scoring.

Apoptosis assay

Apoptosis was measured by counting apoptotic corpses under both control and heat stress conditions, following the methodology described by Lant and Derry (2014)(18). For physiological apoptosis, synchronized L1 larvae were grown until the late L4 stage, and apoptotic corpses were scored 28 hours later. For heat stress-induced apoptosis, worms were subjected to 16 hours of heat stress at 30°C starting at the late L4 stage. Afterward, worms were allowed to recover for 12 hours at 20°C before scoring. Worms were mounted on 3% agar pads, paralyzed with 25 µM levamisole, and analyzed via DIC microscopy using a Zeiss Axio Imager M1.

Microscopy and germline staining

For CHN-1 localization, *chn-1(syb299)* worms were immobilized on 3% agarose pads with 25 µM tetramisole and imaged immediately using an inverted Zeiss LSM800 laser-scanning confocal microscope with a 63× oil immersion objective. mCherry fluorescence was visualized using 561-nm excitation lasers. Brightfield images of germline and gonadal organization were captured using a Nikon SMZ25 microscope with a brightfield filter.

Germline isolation and staining were performed with slight modifications based on the protocol by Lant and Derry (2014) (19). Synchronized adult worms were immobilized in 25 µM levamisole, and germlines were extruded by cutting just below the pharynx or above the tail tip. The germlines were fixed in 2% paraformaldehyde for 10 minutes, sealed with a coverslip, and flash-frozen in liquid nitrogen for 2 minutes. After removing the coverslip, slides were immersed in ice-cold 1:1 acetone:methanol for 1 minute. Germlines were then permeabilized with 1% Triton X-100 in PBS for 10 minutes and blocked with 1% BSA in PBST. Anti-CHN-1 primary antibody (1:100) (20) was applied overnight at 4°C, followed by washing and incubation with Alexa Fluor™ 568 Goat anti-Rabbit secondary antibodies (Invitrogen) for 1

hour. Samples were counterstained with Vectashield antifade mounting medium containing DAPI (Vector Laboratories) and imaged using confocal microscopy.

Proteomics

Proteins were extracted from *C. elegans* pellet into a urea buffer (8 M urea, 100 mM Tris pH 8.5) by sonication on ice for 20 rounds of 2 sec pulses with a sonication probe. The lysates were centrifuged for 2 min at 13,000 × g and supernatants were diluted to 0.4 M urea with 100 mM HEPES pH 8.5. Then the supernatants were digested overnight at 37°C in 100 mM HEPES containing 10 mM tris(2-carboxyethyl)phosphine (TCEP, 15 mM 2-chloroacetamide and trypsin at a protein/enzyme ratio of 100:1. Trifluoroacetric acid (TFA) was added to a final concentration of 1%. Tryptic peptides were labelled using an on-column TMT labeling protocol(21). TMT-labeled samples were compiled into a TMT-16 sample and concentrated. Peptides in the compiled sample were fractionated (8 fractions) using the Pierce™ High pH Reversed-Phase Peptide Fractionation Kit. Prior to liquid chromatography–mass spectrometry (LC-MS) measurement, the peptide fractions were reconstituted in 0.1% TFA, 2% acetonitrile in water. Chromatographic separation was performed on an Easy-Spray Acclaim PepMap column 50 cm long × 75 µm inner diameter at 55°C by applying 120 min acetonitrile gradients in 0.1% aqueous formic acid at a flow rate of 300 nl/min. An UltiMate 3000 nano-LC system was coupled to a Q Exactive HF-X mass spectrometer via an easy-spray source (all Thermo Fisher Scientific). The Q Exactive HF-X was operated in TMT mode with survey scans acquired at a resolution of 60,000 at m/z 200. Up to 15 of the most abundant isotope patterns with charges 2-5 from the survey scan were selected with an isolation window of 0.7 m/z and fragmented by higher-energy collision dissociation (HCD) with normalized collision energies of 32, while the dynamic exclusion was set to 35 s. The maximum ion injection times for the survey and tandem mass spectrometry (MS/MS) scans (acquired with a resolution of 45,000 at m/z 200) were 50 and 96 ms, respectively. The ion target value for MS was set to 3e6 and for MS/MS to 1e5, and the minimum AGC target was set to 1e3. The data were processed with MaxQuant v. 1.6.17.0 (22), and the peptides were identified from the MS/MS spectra searched against UniProt *C. elegans* reference proteome (UP000001940) using the built-in Andromeda search engine. Reporter ion MS2-based quantification was applied with reporter mass tolerance = 0.003 Da and min. reporter PIF = 0.75. Cysteine carbamidomethylation was set as a fixed modification and methionine oxidation, glutamine/asparagine deamination, as

well as protein N-terminal acetylation, were set as variable modifications. For *in silico* digests of the reference proteome, cleavages of arginine or lysine followed by any amino acid were allowed (trypsin/P), and up to two missed cleavages were allowed. The false discovery rate (FDR) was set to 0.01 for peptides, proteins, and sites. A match between runs was enabled. Other parameters were used as pre-set in the software. Unique and razor peptides were used for quantification enabling protein grouping (razor peptides are the peptides uniquely assigned to protein groups and not to individual proteins). Reporter intensity corrected values for protein groups were loaded into Perseus v. 1.6.10.0 (23). Standard filtering steps were applied to clean up each dataset: reverse (matched to decoy database), only identified by site, and potential contaminant (from a list of commonly occurring contaminants included in MaxQuant) protein groups were removed. Reporter intensity corrected values were Log2 transformed. Protein groups with all values were kept. Reporter intensity values were then normalized by median subtraction within TMT channels. Student's t-tests (permutation-based FDR = 0.01, S0 = 0.1) were performed to return protein groups, which levels were statistically significantly changed between the sample groups investigated. The abundance change thresholds of Log2FC > |0.5| was then applied to deliver protein groups with levels deemed reproducibly decreased or increased. This dataset has been deposited to the ProteomeXchange Consortium (24) via the PRIDE partner repository (25) with the dataset identifier PXD048200.

The 25 significantly higher number of downregulated CHN-1 substrates in HSP-1EEYD under heat stress conditions were subjected to SimpleMine analysis, a tool from Wormbase (26). The inputs used were the Wormbase-ID of these genes and the data retrieval parameter was set to all checkboxes. The data retrieved from this analysis are mentioned in Table S5.

Proteomics results analysis

A compilation of substrates and interactors for CHN-1/CHIP was developed based on literature (27–31), as well as interactome databases (32, 33). Both human and *C. elegans* proteins were matched to their corresponding orthologs using OrthoList 2 (34), according to criteria that required consensus from at least two different programs and excluded partial matches. In our methodology, we maintained the integrity of the initial data, specifying the protein (CHN-1 or

CHIP) and the species (*C. elegan*s or *H. sapiens*) from which the information was originally derived.

References

1. Waterhouse, A., Bertoni, M., Bienert, S., Studer, G., Tauriello, G., Gumienny, R., Heer, F. T., De Beer, T. A. P., Rempfer, C., Bordoli, L., Lepore, R., and Schwede, T. (2018) SWISS-MODEL: Homology modelling of protein structures and complexes. *Nucleic Acids Res.* **46**, W296–W303
2. Zhang, M., Windheim, M., Roe, S. M., Peggie, M., Cohen, P., Prodromou, C., and Pearl, L. H. (2005) Chaperoned ubiquitylation - Crystal structures of the CHIP U box E3 ubiquitin ligase and a CHIP-Ubc13-Uev1a complex. *Mol. Cell*. 10.1016/j.molcel.2005.09.023
3. Friesner, R. A., Banks, J. L., Murphy, R. B., Halgren, T. A., Klicic, J. J., Mainz, D. T., Repasky, M. P., Knoll, E. H., Shelley, M., Perry, J. K., Shaw, D. E., Francis, P., and Shenkin, P. S. (2004) Glide: A New Approach for Rapid, Accurate Docking and Scoring. 1. Method and Assessment of Docking Accuracy. *J. Med. Chem.* **47**, 1739–1749
4. Ponder, J. W., and Case, D. A. (2003) Protein Simulations. *Adv. Protein Chem.* **66**, 27–85
5. Salomon-Ferrer, R., Götz, A. W., Poole, D., Le Grand, S., and Walker, R. C. (2013) Routine microsecond molecular dynamics simulations with AMBER on GPUs. 2. Explicit solvent particle mesh ewald. *J. Chem. Theory Comput.* **9**, 3878–3888
6. Tian, C., Kasavajhala, K., Belfon, K. A. A., Raguette, L., Huang, H., Migues, A. N., Bickel, J., Wang, Y., Pincay, J., Wu, Q., and Simmerling, C. (2020) Ff19SB: Amino-Acid-Specific Protein Backbone Parameters Trained against Quantum Mechanics Energy Surfaces in Solution. *J. Chem. Theory Comput.* **16**, 528–552
7. Weiner, P. K., and Kollman, P. A. (1981) AMBER: Assisted model building with energy refinement. A general program for modeling molecules and their interactions. *J. Comput. Chem.* **2**, 287–303
8. Jorgensen, W. L., Chandrasekhar, J., Madura, J. D., Impey, R. W., and Klein, M. L. (1983) Comparison of simple potential functions for simulating liquid water. *J. Chem. Phys.* **79**, 926–935
9. Maier, J. A., Martinez, C., Kasavajhala, K., Wickstrom, L., Hauser, K., Simmerling, C., and Hauser, K. E. (2015) Improving the accuracy of protein side chain and backbone parameters from ff99SB ff14SB: Improving the accuracy of protein side chain and backbone parameters from ff99SB. *Just Accept. Manuscr. • Publ. Date*
10. Andersen, H. C. (1983) Rattle: A “velocity” version of the shake algorithm for molecular dynamics calculations. *J. Comput. Phys.* **52**, 24–34
11. Pedersen, T. D. D. Y. L. Particle mesh Ewald: An N·log(N) method for Ewald sums in large systems
12. Roe, D. R., and Cheatham, T. E. (2013) PTRAJ and CPPTRAJ: Software for Processing and Analysis of Molecular Dynamics Trajectory Data. *J. Chem. Theory Comput.* **9**, 3084–3095
13. Beard, H., Cholleti, A., Pearlman, D., Sherman, W., and Loving, K. A. (2013) Applying physics-based scoring to calculate free energies of binding for single amino acid mutations in protein-protein complexes. *PLoS One*. **8**, 1–11
14. J Comput Chem - 2011 - Michaud-Agrawal - MDAnalysis A toolkit for the analysis of molecular dynamics simulations.pdf
15. Gowers, R., Linke, M., Barnoud, J., Reddy, T., Melo, M., Seyler, S., Domański, J., Dotson, D., Buchoux, S., Kenney, I., and Beckstein, O. (2016) MDAnalysis: A Python Package for the Rapid Analysis of Molecular Dynamics Simulations. *Proc. 15th Python Sci. Conf.* 10.25080/majora-629e541a-00e
16. Genheden, S., and Ryde, U. (2015) The MM/PBSA and MM/GBSA methods to estimate ligand-binding affinities.

Expert Opin. Drug Discov. **10**, 449–461

17. Kamath, R. S., and Ahringer, J. (2003) Genome-wide RNAi screening in Caenorhabditis elegans. *Methods*. **30**, 313–321
18. Lant, B., and Brent Derry, W. (2014) Visualizing apoptosis in embryos and the germline of caenorhabditis elegans. *Cold Spring Harb. Protoc.* **2014**, 278–283
19. Lant, B., and Derry, W. B. (2014) Immunostaining for markers of apoptosis in the caenorhabditis elegans germline. *Cold Spring Harb. Protoc.* **2014**, 510–517
20. Tawo, R., Pokrzywa, W., Kevei, É., Akyuz, M. E., Balaji, V., Adrian, S., Höhfeld, J., and Hoppe, T. (2017) The Ubiquitin Ligase CHIP Integrates Proteostasis and Aging by Regulation of Insulin Receptor Turnover. *Cell*. 10.1016/j.cell.2017.04.003
21. Myers, S. A., Rhoads, A., Cocco, A. R., Peckner, R., Haber, A. L., Schweitzer, L. D., Krug, K., Mani, D. R., Clauser, K. R., Rozenblatt-Rosen, O., Hacohen, N., Regev, A., and Carr, S. A. (2019) Streamlined protocol for deep proteomic profiling of FAC-sorted cells and its application to freshly isolated murine immune cells. *Mol. Cell. Proteomics*. **18**, 995–1009
22. Tyanova, S., Temu, T., and Cox, J. (2016) The MaxQuant computational platform for mass spectrometry-based shotgun proteomics. *Nat. Protoc.* **11**, 2301–2319
23. Tyanova, S., Temu, T., Sinitcyn, P., Carlson, A., Hein, M. Y., Geiger, T., Mann, M., and Cox, J. (2016) The Perseus computational platform for comprehensive analysis of (prote)omics data. *Nat. Methods*. **13**, 731–740
24. Deutsch, E. W., Csordas, A., Sun, Z., Jarnuczak, A., Perez-Riverol, Y., Ternent, T., Campbell, D. S., Bernal-Llinares, M., Okuda, S., Kawano, S., Moritz, R. L., Carver, J. J., Wang, M., Ishihama, Y., Bandeira, N., Hermjakob, H., and Vizcaíno, J. A. (2017) The ProteomeXchange consortium in 2017: Supporting the cultural change in proteomics public data deposition. *Nucleic Acids Res.* **45**, D1100–D1106
25. Perez-Riverol, Y., Bai, J., Bandla, C., García-Seisdedos, D., Hewapathirana, S., Kamatchinathan, S., Kundu, D. J., Prakash, A., Frericks-Zipper, A., Eisenacher, M., Walzer, M., Wang, S., Brazma, A., and Vizcaíno, J. A. (2022) The PRIDE database resources in 2022: A hub for mass spectrometry-based proteomics evidences. *Nucleic Acids Res.* **50**, D543–D552
26. Davis, P., Zarowiecki, M., Arnaboldi, V., Becerra, A., Cain, S., Chan, J., Chen, W. J., Cho, J., da Veiga Beltrame, E., Diamantakis, S., Gao, S., Grigoriadis, D., Grove, C. A., Harris, T. W., Kishore, R., Le, T., Lee, R. Y. N., Luypaert, M., Müller, H. M., Nakamura, C., Nuin, P., Paulini, M., Quinton-Tulloch, M., Raciti, D., Rodgers, F. H., Russell, M., Schindelman, G., Singh, A., Stickland, T., Van Auken, K., Wang, Q., Williams, G., Wright, A. J., Yook, K., Berriman, M., Howe, K. L., Schedl, T., Stein, L., and Sternberg, P. W. (2022) WormBase in 2022-data, processes, and tools for analyzing Caenorhabditis elegans. *Genetics*. 10.1093/genetics/iyac003
27. Das, A., Thapa, P., Santiago, U., Shanmugam, N., Banasiak, K., Dąbrowska, K., Nolte, H., Szulc, N. A., Gathungu, R. M., Cysewski, D., Krüger, M., Dadlez, M., Nowotny, M., Camacho, C. J., Hoppe, T., and Pokrzywa, W. (2022) A heterotypic assembly mechanism regulates CHIP E3 ligase activity . *EMBO J.* **41**, 1–24
28. Balaji, V., Müller, L., Lorenz, R., Kevei, É., Zhang, W. H., Santiago, U., Gebauer, J., Llamas, E., Vilchez, D., Camacho, C. J., Pokrzywa, W., and Hoppe, T. (2022) A dimer-monomer switch controls CHIP-dependent substrate ubiquitylation and processing. *Mol. Cell*. **82**, 3239-3254.e11
29. Mukhopadyay, U., Levantovsky, S., Gharbi, S., Stein, F., Behrends, C., and Bhogaraju, S. (2023) A ubiquitin-specific, proximity-based labeling approach for the identification of ubiquitin ligase substrates. *bioRxiv*
30. Bhuripanyo, K., Wang, Y., Liu, X., Zhou, L., Liu, R., Duong, D., Zhao, B., Bi, Y., Zhou, H., Chen, G., Seyfried, N. T., Chazin, W. J., Kiyokawa, H., and Yin, J. (2018) Identifying the substrate proteins of U-box E3s E4B and CHIP by orthogonal

ubiquitin transfer. *Sci. Adv.* **4**, 1–16

31. Mamun, M. M. Al, Khan, M. R., Zhu, Y., Zhang, Y., Zhou, S., Xu, R., Bukhari, I., Thorne, R. F., Li, J., Zhang, X. D., Liu, G., Chen, S., Wu, M., and Song, X. (2022) Stub1 maintains proteostasis of master transcription factors in embryonic stem cells. *Cell Rep.* **39**, 110919

32. Oughtred, R., Rust, J., Chang, C., Breitkreutz, B. J., Stark, C., Willems, A., Boucher, L., Leung, G., Kolas, N., Zhang, F., Dolma, S., Coulombe-Huntington, J., Chatr-aryamontri, A., Dolinski, K., and Tyers, M. (2021) The BioGRID database: A comprehensive biomedical resource of curated protein, genetic, and chemical interactions. *Protein Sci.* **30**, 187–200

33. Li, Z., Chen, S., Jhong, J. H., Pang, Y., Huang, K. Y., Li, S., and Lee, T. Y. (2021) UbiNet 2.0: A verified, classified, annotated and updated database of E3 ubiquitin ligase-substrate interactions. *Database*. **2021**, 1–10

34. Kim, W., Underwood, R. S., Greenwald, I., and Shaye, D. D. (2018) Ortholist 2: A new comparative genomic analysis of human and caenorhabditis elegans genes. *Genetics*. **210**, 445–461

Supplemental figure S1. A. Plot showing the RMSD of the CHN-1 in complex with GPTIEEV/YD peptides and alone throughout the molecular dynamics simulations. **B.** Plot showing the RMSF of the CHN-1 in complex with GPTIEEV/YD peptides and alone throughout the molecular dynamics simulations. **C.** Superimposed structures of the most frequent EEYD and EEVD peptide conformations in protomer 1 and 2. Conformations were identified via k-means clustering (k=3) over the 100 ns simulation, sampling every 5th frame. EEVD is shown in cyan with V in red, and EEYD in green with Y in orange. **D.** PCA of the CHN-1 in complex with GPTIEEV/YD peptide based on the molecular dynamics simulations.

Supplemental figure S2. A. Survival curve of HSP-1^{EEYD} and *chn-1(by155)*, HSP-1^{EEYD} worms exposed or not to oxidative stress due to paraquat treatment. The figure represents data from 3 biological repeats with n= 135-156 worms per strain per condition. Statistical significance was determined using Mantel-Cox log-rank test; the significance level per *p*-value is shown adjacent to the graph (ns - *p*-value > 0.05). **B.** Representative brightfield images of germline regions in HSP-1^{EEYD} and *chn-1(by155)*, HSP-1^{EEYD} young adult worms exposed or not to HS. **C.** Immunostaining of CHN-1 in excised germlines from the indicated *C. elegans* strains using anti-CHN-1 antibodies. DAPI (blue) highlights the nuclei. **D.** Bar plot showing the number of hatched eggs within the first 24 hours of adulthood at 20°C across indicated worm strains. The graph represents data from 3 biological repeats each containing 18-20 worms per strain per condition. Data were analyzed using 2-way ANOVA. Statistical significance was determined using Tukey's multiple comparisons test; the stars denote the significance level per *p*-value and are shown adjacent to the graph. The results are plotted as the mean ± S.D. **E.** Survival curve of HSP-1^{EEYD} and *chn-1(by155)*, HSP-1^{EEYD} worms exposed or not to HS. The figure represents data from 3 biological repeats with n= 241-272 worms per strain per condition.

Supplemental figure S3. Representative images from 3 independent experiments showing late-pachytene cells in the *C. elegans* germline 12 hours post-heat stress (16 hours at 30°C) for the indicated strains. Arrowheads mark apoptotic corpses. Scale bar: 10 µm.

Supplemental table S1. Results of the EEYD vs. EEVD peptide comparison analysis based on the most frequent conformations identified from clustering the 100 ns molecular dynamics simulations. RMSD was calculated using PyMOL v.2.5.5 (the PyMOL Molecular Graphics System, Version 2.6 Schrödinger, LLC) between aligned peptide backbones. Contact analysis, also performed in PyMOL, identified protein residues in 4 Å proximity to the peptide (between heavy atoms).

**Metric**	**Protomer 1**	**Protomer 2**
**RMSD [Å]**	1.709	3.714
**Protein Residues in Contact**		
**EEYD**	294, 323, 326, 328	12, 15, 16, 37, 38, 229
**EEVD**	294, 297, 323, 324	12, 15, 45, 72, 75, 76, 229
**Centroid Distance [Å]**	11.709	6.970

Supplemental table S2. Binding free energy components for the CHN-1-peptide complexes calculated by MM-GBSA analysis. All energies are in Kcal/mol with standard deviations given in parentheses.

**Peptide variant**	**MM-GBSA**						
	**ΔE_{VDW}**	**ΔE_{ELE}**	**ΔE_{GB}**	**ΔE_{Surf}**	**ΔG_{gas}**	**ΔG_{Sol}**	**ΔG_{bind}**
EEVD	-15.86 (2.54)	-272.60 (43.32)	290.49 (43.67)	-2.81 (0.52)	-288.46 (43.11)	287.67 (43.25)	-0.78 (3.17)
EEYD	-35.94 (3.91)	-323.67 (41.04)	351.09 (38.40)	-5.55 (0.38)	-359.62 (40.84)	345.53 (38.19)	-14.09 (4.62)

ΔE_{VDW} - van der Waals contribution from molecular mechanics; ΔE_{ELE} - electrostatic energy as calculated by the molecular mechanics force field; ΔG_{GB} - electrostatic contribution to the solvation-free energy calculated by the generalized Born method; ΔE_{Surf} - solvent-accessible surface area; ΔG_{gas} - gas phase interaction energy; ΔG_{Sol} - solvation-free energy; ΔG_{bind} - binding-free energy.

Supplemental table S7. *C. elegans* strains used in this study.

Genotype	Identifier	Description
wild type (control)	Bristol - N2 strain	*C. elegans* wild isolate (control)
chn-1(by155)I	BR2823	*chn-1* loss-of-function allele
chn-1(by155)I; unc-119(ed4)III; hhIs136[unc-119(+); chn-1::chn-1::FLAG]	PP1562	*chn-1* overexpression strain
chn-1(syb299)I	PHX299	Strain with CHN-1 tagged with mCherry
hsp-1(syb5159)IV	PHX5159	Strain with V to Y substitution in HSP-1 EEVD motif
hsp-1(syb5159)IV; chn-1(syb299)I	WOP604	Strain with CHN-1 mCherry tag and V to Y substitution in HSP-1 EEVD motif
hsp-1(syb5159)IV; chn-1(by155)I	WOP548	Strain with V to Y substitution in HSP-1 EEVD motif and *chn-1* loss-of-function allele
hsp-1(syb5159)IV; chn-1(by155)I; unc-119(ed4)III; hhIs136[unc-119(+); chn-1::chn-1::FLAG]	WOP547	Strain with V to Y substitution in HSP-1 EEVD motif and *chn-1* overexpression
