# Supplementary material for: HSP70 inhibits CHIP E3 ligase activity to maintain germline function in Caenorhabditis elegans
Source: J Biol Chem. 2024 Oct 9;300(11):107864. doi: 10.1016/j.jbc.2024.107864 (PMC11567022; doi:10.1016/j.jbc.2024.107864)
Supplement: Supplemental Tables Legends [file mmc9.docx]

**Supplemental table 1**: Results of the EEYD vs. EEVD peptide comparison analysis based on the most frequent conformations identified from clustering the 100 ns molecular dynamics simulations.

**Supplemental table 2**: Binding free energy components for the CHN-1-peptide complexes calculated by MM-GBSA analysis.

**Supplemental table 3**: Proteomics dataset of control and HSP-1^EEYD^ strain for normal and HS treated worms.

**Supplemental table 4**: List of CHN-1/CHIP interactors and substrates.

**Supplemental table 5**: Description of 25 proteins, as indicated on Venn diagram in Fig 3B, that were both present in Table S2 and downregulated in control vs. control HS & HSP-1^EEYD^ vs. HSP-1^EEYD^ HS.

**Supplemental table 6**: Raw data of individual lifespan and egg hatching experiments.

**Supplemental table 7**: *C. elegans* strains used in this study.
